# Stress Oximetry: Description of a Test to Determine Readiness for Discontinuing Oxygen Therapy in Infants with Chronic Lung Disease

**DOI:** 10.1155/2018/8151678

**Published:** 2018-09-09

**Authors:** Naveed Hussain, Janet Schwenn, Jennifer Trzaski, Mariann Pappagallo

**Affiliations:** ^1^Division of Neonatology, Department of Pediatrics, Connecticut Children's Medical Center and University of Connecticut School of Medicine, 263 Farmington Avenue, Farmington, CT 06030-2948, USA; ^2^Department of Respiratory Therapy, University of Connecticut Health Center, Farmington, CT, USA; ^3^Division of Neonatology, Department of Pediatrics, Connecticut Children's Medical Center, Hartford, CT 06010, USA; ^4^Division of Neonatology, University of Connecticut Health Center, Farmington, CT 06030, USA

## Abstract

**Background:**

In infants with CLD there are no objective tests to monitor an infant's progress towards weaning out of oxygen inhalation therapy (O_2_IT). A test involving staged maneuvers of increasing respiratory stress while decreasing oxygen support, termed Stress Oximetry (StressOx), has been used at our center for weaning O_2_IT.

**Objective:**

To report the clinical utility of “StressOx” in evaluating readiness for discontinuing O_2_IT in infants with CLD.

**Methods:**

A retrospective review was done of StressOx tests administered at our center from 2002-2008. StressOx was performed based on a consistent clinical protocol in all eligible infants on O_2_IT. O_2_IT was generally discontinued after infant had passed two StressOx tests and subsequently infants were monitored for a minimum of 7 days to determine successful weaning.

**Results:**

There were 279 infants with 899 tests that met inclusion criteria. An average of 3 tests per infant was done, one week apart. The test had a specificity of 97.4% and a positive predictive value of 99.6% in determining success of discontinuing O_2_IT.

**Conclusions:**

StressOx appears to be a clinically useful test that may help in determining an infant's ability to successfully wean out of O_2_IT. Further validation of this test is warranted.

## 1. Introduction

Oxygen inhalation therapy (O_2_IT) is important in the management of chronic lung disease (CLD) in infants [1, 2]. However, increased exposure to oxygen radicals and hyperoxia is implicated in many neonatal diseases [3–5]. Therefore, judicious use of oxygen therapy is needed to ensure optimal outcomes [6, 7]. The decision to discontinue oxygen inhalation therapy is largely subjective and there are no standards regarding acceptable saturation range or adequate periods of observation needed after this change [8, 9]. At the time of discharge a “car seat test” is performed but the reliability of this to predict desaturations in a simulated car ride has been questioned [10]. Furthermore, oxygen saturations are not commonly monitored once the infant is discharged home breathing room air, and any resulting adverse consequences have not been well studied [11–15]. An objective method for assessing an infant's ability to tolerate discontinuing O_2_IT is therefore needed.

Bronchopulmonary dysplasia (BPD) is the most common condition responsible for CLD in infants [16]. Walsh et al. have described and validated a test to help classify BPD based on a standardized physiology-based protocol of oxygen wean at 36 weeks' postmenstrual age (PMA) [17, 18]. But there are no guidelines for determining the best time to wean O_2_IT in an infant with established BPD or CLD [19]. Currently, in most NICUs, when an infant is weaned out of O_2_IT, there are no objective, standardized indicators of success or failure [20]. The length of time for monitoring after discontinuing O_2_IT is also varied, and short or long term consequences of discontinuing O_2_IT have not been reported [15, 21].

A “stress test” may be defined as a structured, step-wise escalation of physiological burden on an organ system in order to safely identify its physiologic reserve or characterize its threshold for decompensation. A well-known example is “exercise testing” for assessment of cardiac function [22]. Similar “stress tests” have been used effectively in detecting latent cardiorespiratory problems in children and adults [23, 24]. However, their use in neonates has not been described.

In premature and newborn infants, the main stressors are crying, activity, and oral feeding [25]. Based on the principle of “stress tests” we have devised a “Stress Oximetry” (StressOx) test that incorporates various infant stressors such as crying and feeding to assess the cardiopulmonary reserve of infants with CLD on O_2_IT. This test has been in use in our NICU for over 2 decades. The aim of this study was to describe StressOx and report its utility in assuring safe and successful discontinuation of O_2_IT prior to discharge in infants with CLD.

## 2. Materials and Methods

This was a single-center retrospective study at the University of Connecticut Health Center (UCHC) in Farmington. Inclusion criteria were availability of complete data for infants at ≥34 weeks' postmenstrual age (PMA) who were on O_2_IT for ≥ 14 days with CLD of any cause [19]. Infants with oxygen requirements due to congenital heart disease, neurologic conditions, complex genetic syndromes, and chromosomal anomalies were excluded. This study was approved by the UCHC Institutional Review Board.

### 2.1. Patients

A protocol-based approach to StressOx testing has been in use in this NICU for over 2 decades for all infants who met the following criteria: (i) PMA of ≥34 weeks; (ii) stable on O_2_IT for CLD with nasal cannula flow rates ≥ 0.5L/min to ≤ 2 L/min with oxygen concentration ranging from 21% to 100%; (iii) taking a minimum of 15 ml/kg with each oral feeding and showing appropriate growth in the preceding week. Oral feeds during the test were with bottle with expressed mother's milk or formula as appropriate for that infant. Infants' were tested weekly when they met the above criteria. Tests were stopped when the infant passed StressOx test, usually 2 consecutive passes 1 week apart.

### 2.2. Test Personnel and Reports

All tests were performed by one of two trained personnel. Findings from each StressOx test were reported using a standardized form. The attending neonatologist interpreted the results based on predefined criteria and made clinical decisions regarding O_2_IT.

### 2.3. Equipment and Setup

A Nellcor OxiMax N-600 (Nellcor Puritan Bennett Inc., Pleasanton, CA) pulse oximeter and a cardiorespiratory monitor were used to perform StressOx tests. Oximeter limits set were (i) heart rate between 80 and 210 bpm; (ii) SpO_2_ between 90 and 100%; (iii) alarm limit at 50 “Sat seconds” where “Sat seconds” is an averaging algorithm in the monitor that takes into account the percent oxygen saturation drop below a threshold along with the time (seconds) it is breached (Operators' Manual OxiMax N-600, pp 141-144, 2005). It is important to note that the SpO_2_ set of 90-100% was only for the purpose of the test and was not the target for routine care. The target SpO_2_ for routine care was 90-95%.

### 2.4. StressOx Procedure (Figure 1)

At the outset, fraction of inspired oxygen (FiO_2)_ and nasal cannula flow rates were documented.* Baseline*. Procedure began with baseline observations at rest for vital signs, oxygen saturations, and work of breathing on current respiratory support (FiO_2_, nasal cannula). Three staged maneuvers were then performed in sequence.* Stage 1.* On baseline FiO_2_ and cannula flow the infant was stimulated to active/crying state followed by recovery for a minimum of 2 minutes. Test parameters were recorded.* Stage 2.* Respiratory support was completely discontinued and test parameters were recorded while the infant remained in the quiet awake state in room air for a minimum period of 5 minutes. The infant was then stimulated to an active/crying state followed by recovery within a 2 minute period and test parameters were recorded.* Stage 3.* In the final stage, while still without respiratory support, the infant was offered an oral sucking feed by breast or bottle and observed until completion of the feed and test parameters were recorded. The test was continued as tolerated until the infant successfully went through all the three maneuvers. At any time if the infant had drop in heart rate < 80 bpm, significant increase in work of breathing, or persistent (>1 minute) desaturations (SpO_2_ <90%), the test was discontinued.

### 2.5. Documentation of Test Parameters

During all 3 stages, heart rate, respiratory rate, color-change, and SpO_2_ were noted in real-time approximately every 30 seconds. Work of breathing was documented based on respiratory rate, retractions, nasal flaring, accessory muscle use, or head bobbing. A “*desaturation*” was recorded starting at a point where SpO_2_ fell below 90%. A* “desaturation with quick recovery”* was SpO_2_ <90% for <1 minute. A* “significant desaturation”* was SpO_2_ <90% for ≥1 minute. Thresholds for feeding behavior were based on ability to take a minimum of 15 ml/kg/feed within 30 minutes. However, signs of discoordination, especially frequent choking, gagging, or need for additional supports for feeding such as pacing or change in position, were also taken into account. Data gathered for each parameter (saturations, respiratory rate, heart rate, work of breathing, etc.) during each stage were recorded in a standardized report form as the highest and lowest values along with the period of time a parameter was outside predetermined thresholds. Since infants were awake during this test apnea was not used as an assessment parameter.

### 2.6. Rationale for Threshold Criteria Used in the Test

The thresholds and cutoffs for the various parameters used in this test were derived from previously defined norms and have a scientific rational basis. The threshold SpO2 of <90% was chosen for the following reasons: (a) SpO_2_ is not normally distributed and in normal infants <4% of time is spent at <90% SpO_2_ [1, 14]; (b) SpO2 of <90% is to be avoided in infants with CLD in order to prevent complications [26–29].; (c) SpO2 of >=90% is used by most centers as the threshold for passing for room air challenge tests [30–32]. The averaging time settings of an oximeter are important because a longer averaging time (16-20 sec) will not detect desaturation dips but a very short averaging time (<4 sec) will give more alarms [21]. We chose an averaging time of 10 seconds to avoid both problems. The use of “Sat seconds” algorithm available in most oximeters is another useful strategy in decreasing irrelevant monitor alarms.

### 2.7. Interpretation of Findings

Interpretation of the test was done by the attending neonatologist using predetermined criteria based on the documented data on test report forms. The criteria were determined based on general consensus after review of literature [20, 25, 28, 33–40].* Criteria for failing StressOx test were as follows:* (i)* “significant desaturation”* with SpO_2_ <90% for ≥1 minute; (ii) >5* “desaturations with quick recovery”* along with two additional respiratory symptoms (increased work of breathing, tachypnea >80 breaths/ minute) or inability to take in appropriate feed volume; or (iii) for infants taking >15 ml/kg/feed as pretest baseline, inability to complete the same volume during a 30-minute feeding time in room air.* Criteria for passing StressOx test were as follows*: (i) maintained SpO_2_ ≥90%, with no significant change in work of breathing or (ii) having <5* “desaturation with quick recovery”* with up to one additional respiratory symptom (work of breathing or tachypnea). In either case, respiratory performance had to be accompanied by adequate feeding as demonstrated by ability to take oral feeds of ≥15 ml/kg/feed or pretest baseline volume.

### 2.8. Frequency of the Test and Follow-Up

Once an infant met criteria for testing, StressOx was performed weekly until the infant passed the test and was taken out of O_2_IT or was discharged on oxygen. Infants who failed the test were returned to their baseline level of respiratory support. In most infants, once they passed the StressOx test for two consecutive weeks, respiratory support was discontinued and the infant was further monitored for a minimum of 7 days in the hospital to observe SpO_2_, feed intake, and growth before discharging home. Any infant that subsequently showed significant desaturations (SpO_2_ <90% for >1 minute), poor oral intake (<120 ml/kg/d), or poor growth (<15 g/day) was returned to previous level of respiratory support with O_2_IT and weekly testing was restarted. In some instances, based on performance on StressOx, changes in respiratory support and medical management were made.* Criteria for discharge home without O*_2_*IT* included absence of significant desaturations for 7 days after passing StressOx and discontinuation of O_2_IT, adequate oral intake to maintain age appropriate weight gain, absence of apnea episode for 7 days, and ability to regulate body temperature in an open crib. The local NICU definitions of hypoxemia and need for O_2_IT did not change during the study period.

### 2.9. Test Sequences

In most instances, a passed test followed by a failed test would have a repeat StressOx done until two consecutive pass tests. The outcome of oxygen-need after two passed tests was used to categorize true and false positive test.

### 2.10. Interrater Reliability

Tests were randomly selected to be done in the presence of both observers and their independent scores were reviewed for agreement of findings related to pass or fail test results based on observation data from StressOx test documented on standardized forms. Interrater reliability was measured using the* kappa* score.

## 3. Results

### 3.1. StressOx Tests Done

**Figure 2:**
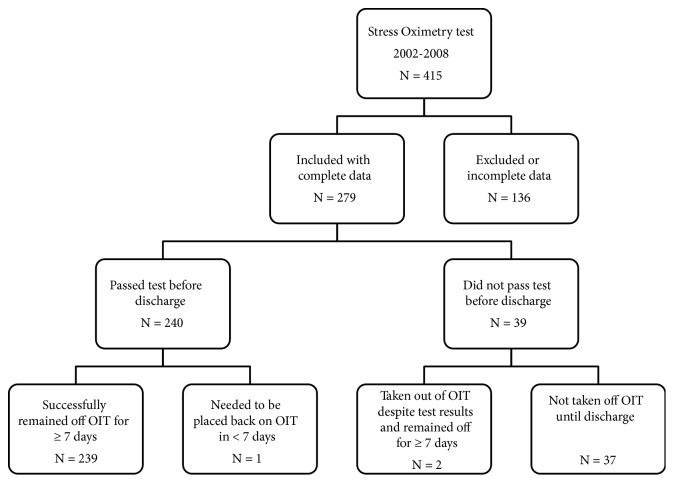
Patients with “Stress Oximetry Test”.

Of 3,349 admissions to the NICU between 2002 and 2008, StressOx was administered to 415 infants for a total of 1,545 tests. After applying exclusion criteria, 279 infants and 899 tests were analyzed (Figure 2). Of the 240 infants who passed the test, 239 remained off need for O_2_IT and only 1 needed to be placed back within 7 days. Of the 39 infants that did not pass the test before discharge, 2 were taken off despite test results and remained off for > 7 days. The 37 that did not pass the test before discharge were sent home on supplemental oxygen and close follow-up with pulmonologist. A modified StressOx was used by the pulmonologist in the outpatient clinic for determining ongoing need for O_2_IT.

### 3.2. Patient Characteristics

Patient characteristics are shown in Table 1. The personnel-time needed for a single test was ≈40 minutes and infants had a mean of 3 tests prior to discharge. There was no difference in test use between infants based on race, sex, ethnicity or plurality of gestation. The majority (n=266, 95%) of infants had CLD because of prematurity and BPD. However a notable minority (n=13, 5%) of infants were mature infants with CLD secondary to meconium aspiration, pneumonia, or sepsis.

### 3.3. Specificity and Positive Predictive Value

Majority of infants were successfully weaned off oxygen prior to discharge based on the result of StressOx test. Needing oxygen therapy after two successful passes was very unusual and discontinuation of oxygen despite failing the test was only done in only 2 infants. The final decision for discontinuation of O_2_IT was based on the last StressOx test performed and we used the results of this test to determine sensitivity, specificity, negative predictive value, and positive predictive value (Table 2).

### 3.4. Interrater Reliability of the Test

Based on 20 random tests that were observed by both personnel, there was agreement among the two in 19 tests (95%). The kappa score for interrater reliability was 0.9.

### 3.5. Adverse Effects of the Test

Various maneuvers of the test are part of routine care of the hospitalized infant. There were 1,545 tests administered during the study period with no adverse consequences directly related to the test. The state of high activity or crying that was used in the performance of this test was mostly induced by routine infant-care maneuvers such as taking temperature, cleaning or changing clothes or diapers.

## 4. Discussion

The Stress Oximetry Test described in this paper is a screening test used to identify the ability of infants with CLD to wean from O_2_IT and thrive. It requires only routine bedside care-equipment and can easily be performed by NICU personnel. StressOx was safe and highly specific, the interobserver reliability was high, and the test was useful clinically.

Use of O_2_IT is closely related to the diagnosis and treatment of CLD in infants [41]. Despite its central role in diagnosis and management of BPD there is very little information on criteria for discontinuing O_2_IT or the parameters that need to be monitored to ensure the safety and success of this intervention. Duration of oxygen therapy was first used to diagnose BPD by Northway [42, 43] and has since been essential in other suggested modifications of criteria for this diagnosis [19, 44, 45]. A physiologic definition of BPD at 36 weeks' PMA using a step-wise decrease in oxygen supplementation has been standardized and validated by Walsh [17, 18]. It is important to distinguish StressOx test from the test proposed by Walsh for the physiologic definition of BPD. The test by Walsh is primarily a diagnostic test designed to diagnose and categorize BPD, whereas StressOx is a prognosticating tool designed to help with weaning and discontinuation of O_2_IT in patients with previously diagnosed BPD or CLD [17, 18]. Walsh's test refers primarily to oxygen supplementation levels and levels of saturations in the infant without reference to the infant's state of activity or feeding, but StressOx refers to a combination of cardiorespiratory signs along with the infant's state of activity especially feeding and crying.

Other attempts to develop objective criteria for weaning from O_2_IT have not been successful. In a pilot study with 17 subjects, a room air challenge was proposed by Simoes based on SpO_2_ at 40 minutes after discontinuing supplemental oxygen [46]. Other similar approaches have been advocated in which oxygen was discontinued for up to 30 minutes to document that a nadir SpO_2_ value does not fall below 80% [31]. A hypoxia challenge test has been described using subnormal (0.15 FiO2) oxygen and a body plethysmograph but the clinical utility of such a test is impractical in the NICU [47]. Another approach was using modified polysomnography to determine “stable versus unstable” infants but this study with 30 subjects does not provide enough evidence for its regular use [15]. Therefore the aforementioned strategies have not found use in the NICU and there have been no reports on their utility in predicting successful discontinuation of O_2_IT.

In clinical practice, successful weaning of O_2_IT in infants with CLD is determined not only by the infant's ability to maintain oxygen saturations in an “acceptable” range for a “sufficient” period of time, but also by the ability to feed and demonstrate optimal growth. Furthermore, there is great variability in the SpO_2_ values considered “acceptable” in US NICUs [48, 49]. The period of time that an infant is monitored after discontinuing oxygen is similarly variable. There is scarce information on how discontinuation of O_2_IT affects subsequent feeding intake and growth. The ability to ingest an adequate amount of oral feeds is closely linked to the ability of an infant to thrive off O_2_IT [25, 34]. Infants with CLD who are well oxygenated at rest may have unsuspected desaturations after discontinuation of caffeine therapy or during oral feeding and other activities [39, 40, 50]. Infants with CLD also have difficulty in coordinating suck, swallow, and breathing [34, 36]. Feeding by mouth may be the most strenuous task undertaken by the newborn infant and is a good indicator of an infant's respiratory reserve [35, 51]. Choking during feeds may indeed be a sign of respiratory difficulty in an infant [52]. Crying and feeding are the main stressors triggering desaturations in an infant with insufficient pulmonary reserve [12, 37, 38]. Therefore StressOx test provides a more comprehensive assessment of supplemental oxygen needs than methods not incorporating feeding as a key determinant.

There are certain limitations of this study. This report of our current practice is based on a retrospective evaluation of previously collected data on standard forms. Inferences should therefore be drawn with caution. All infants who failed the StressOx test remained on O_2_IT until the next weekly test. It is possible that some of these infants did not continue to require O_2_IT; however this could not be ascertained because these infants were not tried without O_2_IT until the next test. Another potential limitation relates to subjectivity in interpretation of clinical tests. With the StressOx test we have tried to minimize subjectivity by using a standardized data collection form completed by trained personnel and consistently used criteria for test pass/fail with independent interpretation by a neonatologist. Moreover, since oral feeding is an important constituent of the test, problems related solely to feeding anatomy, coordination or maturity unrelated to lung function may have affected the results.

A criticism or limitation of applicability of the weekly tests may be the potential increase in hospital length of stay. In practice however, that has not been the case in this select group of infants who by definition have moderate to severe BPD (need for oxygen therapy at approximately 36 weeks' postmenstrual age) [19]. Another factor that prolongs hospital stay in these infants is ability to take adequate oral feeds. Since feeding ability is part of evaluation in the StressOx test, we believe that both feeding and respiratory status are simultaneously addressed and therefore there is not additional delay in hospital discharge. However, it is possible that some infants may not need two consecutive passes and the quality of performance during the test may be an important predictor. Realizing this, we have been scoring the quality of the responses and plan to report these findings in the future.

## 5. Conclusion

Despite the wide use of O_2_IT in the NICU and the recognition of its potential for overtreatment [53, 54], there are currently no standardized procedures to assess the optimal conditions for its safe discontinuation. The StressOx test is an assessment tool that may provide neonatal caregivers, facing decisions regarding oxygen management in infants with CLD, with additional objective data. Further validation of this test is warranted.

## Figures and Tables

**Figure 1:**
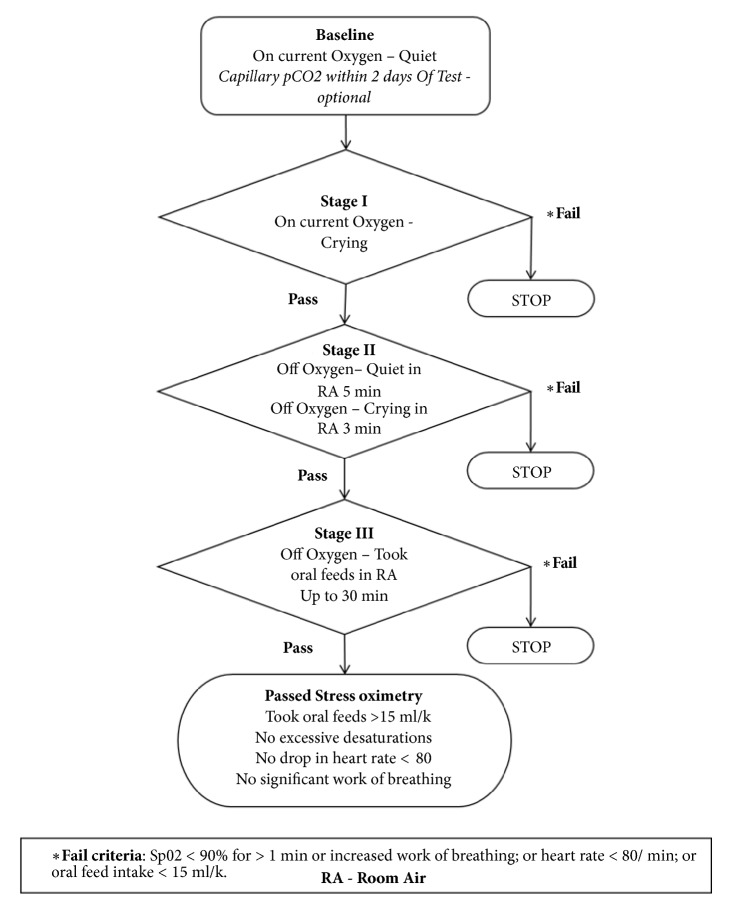
Schema for Stress Oximetry Test.

**Table 1:** Patient Characteristics of infants undergoing StressOx test.

	**Mean (SD)**	**Range**
**Gestational age at birth (wks.)**	28.1 (3.5)	22 - 41
**Birth Weight (gm)**	1200 (710)	435 - 4458
**Age at 1st test (days)**	60 (23)	15 - 169
**Postmenstrual age at **1^**st**^** test (wks.)**	36.8 (2.3)	34.0 - 50.1
**Number of tests per patient**	3.3 (1.8)	1 - 9
**Age at last test (days)**	78 (30)	15 - 225
**Postmenstrual age at last test (wks.)**	39.2 (2.8)	34.0 - 55.1
**Nasal cannula O**_**2**_ ** flow at last test (L/min)**	1 (0.5)	0.25 - 3.0
**FiO**_**2**_ ** at last test **	0.52 (0.27)	0.21 - 1.0
**pCO**_**2**_ ** at last test**	48.2 (5.6)	32.6 - 66.5
**Weight at last test (gm)**	2111 (545)	1190 - 4720

Above data are based on 279 patients and 899 tests from years 2002 to 2008.

**Table 2:** Ability of StressOx test to determine need for oxygen inhalation therapy (O_2_IT).

		Able to come out of O_2_IT and thrive as indicated by extended inpatient monitoring	
		Yes N= 241	No N = 38
StressOx Test	Passed (N =240)	239	1
	Failed (N = 39)	2	37

Total 279 patients studied. Two infants who failed StressOx and yet were able to come out of O_2_IT had no respiratory compromise during the test but failed due to choking episodes during the feed resulting in inability to complete the required amount of feed.

Sensitivity: 239/ (239+2) = 99.2%.

Specificity: 37/ (37+1) = 97.4%.

Positive predictive value: 239/ (239 + 1) = 99.6%.

Negative predictive value: 37/ (37+2) = 94.9%.

## Data Availability

Data will be made available by the corresponding author upon request and after approval of the Institutional Review Board.

## Abbreviations

BPD: Bronchopulmonary dysplasia
CLD: Chronic lung disease
NICU: Neonatal Intensive Care Unit
O_2_IT: Oxygen inhalation therapy
StressOx: Stress oximetry
PMA: Postmenstrual age.
